# Cool executive functions and their association with body mass & fatness and the FTO gene in school-aged children

**DOI:** 10.1038/s41598-023-38808-0

**Published:** 2023-07-27

**Authors:** Paula Szcześniewska, Ewa Bryl, Agata Dutkiewicz, Aneta R. Borkowska, Karolina Bilska, Elżbieta Paszyńska, Agnieszka Słopień, Monika Dmitrzak-Węglarz, Tomasz Hanć

**Affiliations:** 1 Institute of Biology and Human Evolution, Faculty of Biology, Adam Mickiewicz University, 61-614 Poznan, Poland; 2 Department of Child and Adolescent Psychiatry, Poznan University of Medical Sciences, 60-572 Poznan, Poland; 3 Faculty of Education and Psychology, Maria Curie-Sklodowska University, 20-400 Lublin, Poland; 4 Department of Psychiatric Genetics, Department of Psychiatry, Poznan University of Medical Sciences, 61-701 Poznan, Poland; 5 Department of Integrated Dentistry, Poznan University of Medical Sciences, 60-812 Poznan, Poland

**Keywords:** Paediatric research, Cognitive neuroscience

## Abstract

The FTO gene rs9936909 polymorphism is one of the well-documented single nucleotide polymorphisms in the context of increased risk of obesity, including in children. Few studies have tested the association of the FTO gene with cognitive functions. Deficits of “cool” executive functions (EFs) are considered a potential risk factor for excessive weight. The aims of our study were to investigate whether cool EFs are associated with the Body Mass Index, the Fat Mass Index and the risk of excess body mass and overfatness in neurotypically school-aged children, and whether the FTO gene polymorphism is involved in development of this possible association. The sample consisted of 553 children aged 6–12 years old. A body composition analysis, a neuropsychological assessment of EFs, and FTO polymorphism genotyping were performed in the children studied. The study found a significant association of an interference effect in theStroop Color-Word Interference Task and the risk of excessive body fatness, but not excessive body mass. There were no explicit associations between the FTO genotype and EFs deficits. Environmental factors, and particularly low maternal education, appeared to be the strongest contributors to the increased risk of obesity.

## Introduction

A growing number of children and adolescents worldwide are struggling with being overweight or having obesity^1^. The occurrence of obesity in childhood significantly increases the risk of being obese in adulthood and thus the obesity-related negative health consequences^2^. The etiopathogenesis of obesity is complex. The question of why some people are more prone to obesity is multifaceted. Among others, factors such as low socioeconomic status, psychosocial problems, endocrine disruption and, finally, genetic background contribute to obesity^3^. Among the genes influencing fat distribution and body weight is fat mass and the obesity associated gene (FTO)^4^.

The FTO gene, located on chromosome 16 (16q12.2), is thought to encode the enzyme 2-oxoglutarate-dependent nucleic acid demethylase, which expresses itself mostly in pancreatic islet cells and hypothalamus, suggesting its involvement in the regulation of the body’s energy homeostasis^5,6^. There are two allelic variants of the rs9936909 polymorphism (position: chr16:53786615(GRCh38.p14))A and T. Carrying the A allele has been shown to be associated with a higher BMI, higher risk of obesity and a higher risk of excessive body fatness^7^. This association is also significant in children and adolescents^8^, although the rs9939609 polymorphism does not affect birth weight or resting metabolism^9^.

There are various mechanisms proposed to explain the link between FTO and obesity, among others its role in regulating energy homeostasis, regulating the appetite and food intake^10,11^ directly by adipocyte or indirectly through hypothalamic expression^7^. The one of interesting possible explanation is the association of the FTO gene with obesogenic behaviors such as high dietary intake and low physical activity^9,11,12^. Expression of the FTO gene in hypothalamus, cerebellum and cerebral cortex, suggest that this gene may be related to a behavioral phenotype predisposing to obesity through more impulsive behavior and deficits in self-control processes^13^.

From the perspective of neuropsychology human behavior is subordinated to executive functions (EFs). EFs are a range of higher-order cognitive processes that enable goal-oriented behavior^14^. The processes of EFs are often described as having cool and hot components^15^. Cool EFs are activated when solving difficult, novel problems^16^. They engage critical analyses and logic^15^. It includes three main cognitive functions: (1) working memory, (2) inhibitory control and (3) shifting^17^. Hot EFs, in turn, are processes driven by emotion and motivation^15^. The various directions of the relationship between EFs and obesity are analyzed. Both the potential impact of EFs on obesity and obesity on cognitive functioning are also considered^18–20^. The focus of our particular interest is EFs—body mass direction. EFs deficits may occur as difficulties in suppressing automatic impulses to eat tasty, high calorie foods^21^, difficulties to maintain active long-term goals such as healthy eating in the memory^20^ rigid food behaviors and low flexibility in the process of dieting, which result in the more frequent loss of dietary control^22^. However, despite the growing amount of research into the relationship between EFs and body mass, their results are ambiguous^16,18^. Thus, there is a need for further studies in the field of cognitive components playing the role in being overweight or obese.

So far, only a few publications have tested the association of FTO with cognitive functions or the nervous system^23–27^. These publications have generally focused on the elderly, Alzheimer's disease, and depression. Furthermore, previous studies on the EF-obesity relationship have mostly used the Body Mass Index (BMI) as an indicator of obesity—only a few papers include additional indices in children^28^. Since excess body fat may appear before obesity develops, the use of additional indicators of nutritional status appears to be a promising research direction. Thus, the aims of our study were to investigate whether cool EFs are associated with the risk of overfatness and excess body mass, and whether the FTO gene polymorphism is involved in the development of this possible association. However, unlike most studies on the EFs-body mass relationship, we included additional nutritional status indicators such us Fat Mass Index (FMI) and body fatness (%).

## Methods

### The procedure

The work described has been carried out in accordance with The Code of Ethics of the World Medical Association (Declaration of Helsinki) for experiments involving humans and approved by the Institutional Bioethics Board of Poznan University of Medical Sciences (approval no. 542/14). The recruitment for the study was conducted in primary schools located in Poznań, Poland. The criterion for inclusion in the study was the age of 6 to 12 years old and the absence of a formal diagnosis of a mental disorder declared by parents. In order to avoid the potential influence of additional biological and medical factors on executive functioning and body mass, the following exclusion criteria were applied: organic CNS dysfunctions (e.g., epilepsy), endocrine diseases (e.g., Cushing’s syndrome, growth hormone deficiency, hypothyroidism), and current pharmacotherapy. Both the children and their legal guardians were fully informed about the research procedures. The legal guardians gave written consent for theirchildren to participate in the study. The data collection was scheduled between 8 a.m. and 12 p.m. in the children’s schools. All participants in the study were Caucasian.

### Anthropometric assessment and excess weight diagnosis

Body weight measurement and body composition analysis (body fat) were carried out with the use of the multi frequency segmental body composition analyzer TANITA MC-780, which is based on a Bioelectrical Impendence Analysis (BIA). Body height was measured using the anthropometer Seca 213, with a measurement accuracy of ± 1 mm. BMI was calculated and based on the height and weight measurement and was adjusted for sex and age based on the WHO child growth standards with the use of the WHO AnthroPlus software^29^. Excess weight and obesity were diagnosed with the International Obesity Task-Force criteria based on the children’s BMI^30,31^. The body-fat (%) was adjusted for sex and age based on the data of the sample. The Fat Mass Index (FMI)^32^ was calculated on the bases of the height and body fat measurement. The results were standardized by age and sex. The body fat status was diagnosed based on McCarthy centile curves for body fat^33^.

### Cool executive functions assessment

To assess cool EFs in children, the following tests were applied: The Continuous Performance Task (CPT), the Stroop-Color Word Interference Task (SCWT) and the Trail Making Test A and B (TMT A and B). All of the test scores were standardized by age and sex.

#### The continuous performance task (CPT)

The Continuous Performance Task is a computer-based method that assesses the ability to *sustain attention* and attention deficits. The subject's task is to make a motor response to critical stimulus. In other situations, the participant must refrain from reacting. The first version of the test was constructed by Rosvold and colleagues in 1956^34^. In our version of the test, developed in Poland in 2007^35^, the child's task was to press a computer mouse in response to the letter X appearing after the letter A (sequence). At other times the child was asked not to react. In this task it is possible to make two types of errors: omissions (not pressing the button when a stimulus appears) and commissions (pressing the mouse despite the absence of a stimulus). The duration of the task was 15 min. The following indicators were applied: correct reaction time, number of omissions (sustain attention indicator), number of commissions (impulsivity indicator).

#### The stroop color-word interference task (SCWT)

The SCWT is one of the methods for assessing *inhibitory control*. The SCWT version applied in the study consisted of the following 3 parts.

1. *Naming colors.* In this part the speed of naming colors was measured. On a white A4 sheetof paper, five columns and twenty rows were placed with crosses printed in three random colors: red, green, blue. The subject's task was to name all the colors in the fastest possible time.
2. *Reading the names of the colors*. On a white sheet of A4 paper, five columns and twenty rows of randomly selected words were printed—the names of three colors: red, blue, and green. The colors were printed in black ink.
3. *Interference task*. In this part naming colors in a situation of incongruence between the meaning of a word and the color of the font used was measured. On a white sheet of A4 paper there were five columns and twenty rows of words which were the names of the colors: red, blue,and green. The words were written in colored ink, but different from the name of the color, e.g., the word "red" was printed in green. The subject's task was to name the colors of the font, not to read the words. Two indicators of interference were applied:
  1. time interference (the difference between the time of performance in the interference task and the time of performance in the color naming task)
  2. error interference (the difference between the number of errors in the interference task and the number of errors in the color naming task).

#### The trail making test (TMT)

The TMT A and B is a neuropsychological tool for measuring *cognitive flexibility and shifting*^36^. A standard version of the test was applied. The measure of shifting and cognitive flexibility in this task was the ratio of Part B time performance to Part A time performance^36^. As additional indicators the number of errors in part A and B were measured.

#### SNP selection and genotyping

The FTO gene and its polymorphism was selected and based on their previously established relationship with vulnerability to obesity according to the PubMed data base. Genomic DNA (gDNA) was extracted from salivary samples using the Oragene®-DNA protocol^37^. We genotyped one polymorphism of the fat mass and obesity-associated gene (rs 9939609, A > T). The selected polymorphism was genotyped using the TaqMan single-nucleotide polymorphism (SNP) allelic discrimination method with the ABI 7900HT system (Applied Biosystems).

### Controlled variables

#### Socioeconomic status and parental education

To assess the socioeconomic status of the participants, the children’s legal guardians completed a questionnaire containing questions about the parent’s place of residence, the parental subjective assessment of the socioeconomic situation of the family, and the parents' level of education—both mothers and fathers. The parents' educational assessment was based on their level of formal education: primary, vocational, secondary, or higher (bachelor's/master's degree). The size of the place of residence was assessed using the number of residents: ‘village’, small cities: ‘city up to 10,000 residents’ and ‘city with 10,000–50,000 residents’, average city: ‘city with 50,000–100,000 of residents’, and large cities: ‘city with 100,000–500,000 residents’ and ‘city above 500,000 residents’. Since the direct question about income levels are often difficult for parents, the question about the parental subjective assessment of the socioeconomic situation of the family was implied instead. For the statistical analysis, the answers were divided into three categories: ‘bad or very bad’, ‘average’ and ‘good or very good’ families’ material status.

#### Mother’s BMI

The mothers of the examined children were additionally asked to fill in a questionnaire about themselves with anthropometric data: current body mass and body height. Based on the calculated mother’s BMI, we classified their body mass according to WHO standards, consecutively: underweight (BMI < 18.5), healthy weight (BMI 18.5–24.99), overweight (BMI 25.0–29.99) and obesity (BMI > 30). The hypothesis. We have put forward the following hypothesis:

1. Poor cool EFs are related to overweight/obesity diagnosed according to IOTF criteria
2. Poor cool EFs are related to overfatness/obesity diagnosed according to the McCarthy criteria
3. Cool EFs are associated with the children’s BMI and FMI
4. The FTO gene is associated with the children’s cool EFs
5. There is an interaction between the FTO gene, cool EFs and obesity in children.

### Statistical analysis

All the analyses were performed using IBM SPSS Statistics 28.0. All of the statistical tests were considered to be statistically significant at *p* < 0.05 and not corrected for multiple testing.

Although it was not possible to collect a full set of data for every child, we decided not to exclude the participants with incomplete data to keep as large a group as possible in the analysis.

#### Cool executive functions—cluster analysis

Due to the large number of indicators of executive functioning, we conducted a cluster analysis using the k-means method based on standardized data.

#### Logistic regression analysis

In order to assess: (1) the risk of excess body weight and excess body fatness in relation to cool EFs clusters, inhibitory control, sustained attention and shifting; (2) the risk of lower executive functioning in relation to the FTO genotype; (3) the role of controlled variables in the risk of overweight, overfatness and obesity; (4) and the role of the FTO genotype in the risk of overweight and obesity, including cool EFs clusters as a controlled variable; adjusted and unadjusted logistic regression analyses were performed. For all logistic regression analyses that included the EFs results, the z-scores executive function tests (CPT, TMT, SCWT) variables were dichotomized and based on a cluster analysis. The FTO genotype variable has been categorized twice: (1) children with allele risk, AA + AT genotype, vs children with no allele risk, TT genotype and (2) homozygotes AA vs homozygotes TT.

#### Correlation analysis

To assess the correlations between EFs and BMI, FMI, body fat (%) z-scores, the Pearson correlation analysis was applied.

#### ANOVA analysis

To assess the differences in neuropsychological parameters between children according to their FTO genotype (AA, AT, TT), the ANOVA analysis was applied. A factorial ANOVA was used to answer the research question of whether there is an interaction between FTO genotype (AA, AT, TT) and overall EFs (clusters) for the BMI and FMI z-scores of the children studied.

## Results

540 children, including 47.6% girls and 52.4% boys took apart in our study. The largest subsets were children with healthy body mass. The percentage of obese children according to the IOTF criteria was above 5%, however, when diagnosed by the McCarthy criteria, the result was 20%. Most of the children came from large cities and well-educated families, with at least one parent with higher education. The detailed subject characteristics with descriptive statistics are detailed in Table 1 below.

**Table 1:** Characteristics of the sample (frequencies).

Child characteristics	The information available for	n (%)
Sex	N = 540	
Girls		257 (47.6%)
Boys		283 (52.4%)
Weight status (IOTF criteria)	N = 540	
Underweight		56 (10.4%)
Healthy weight		371 (68.7%)
Overweight		85 (15.7%)
Obesity		28 (5.2%)
Body fat status (McCarthy criteria)	N = 534	
Underfat		3 (0.6%)
Healthy		338 (63.3%)
Overfat		86 (16.1%)
Obesity		107 (20.0%)
FTO genotype	N = 458	
TT		148 (32.3%)
AT		212 (46.3%)
AA		98 (21.4%)
Family characteristics		
Mother’s weight status (WHO criteria)	N = 521	
Underweight		19 (3.6%)
Healthy weight		354 (67.9%)
Overweight		105 (20.2%)
Obesity		43 (8.3%)
Mothers’ education level	N = 528	
Primary		5 (0.9%)
Vocational		53 (10.0%)
Secondary		126 (23.9%)
Higher		344 (65.2%)
Fathers’ education level	N = 509	
Primary		15 (2.9%)
Vocational		97 (19.1%)
Secondary		146 (28.7%)
Higher		251 (49.3%)
The place of residence	N = 513	
Village		41 (8.0%)
City with up to 10,000		3 (0.6%)
City with 10,000. up to 50,000. residents		43 (8.4%)
City with 50,000up to 100,000. residents		17 (3.3%)
City with 100,000 up to 500,000. residents		37 (7.2%)
City with over 500,000 residents		372 (72.5%)
Parental assessment of socioeconomic situation of the family	N = 517	
Good or very good		407 (78.7%)
Average		91 (17.6%)
Bad or very bad		19 (3.7%)

*IOTF* International Obesity Task Force criteria, *N* number of individuals for whom the data were available, *n* number of individuals in the categories, *%* percent of the sample.

### Executive functioning and anthropometric indicators

The results of the cluster analysis allowed us to divide the children studied into 2 groups: higher and lower functioning in terms of executive function. Details of the cluster analysis are presented in Table 2 below. In addition, we attached raw neuropsychological test results according to age and sex.in the supplementary materials (Table S1).

**Table 2:** Executive functions—cluster analysis (z-scores).

EF indicator	Clusters (mean ± SD)	
	Cluster 1 (lower scores) N = 89	Cluster 2 (higher scores) N = 345
CPT: reaction time (z-scores)	.21(1.33)	–.08 (.94)
CPT: commission errors (z-scores)	.91 (1.45)	–.21 (.70)
CPT: omission errors (z-scores)	.44 (1.25)	0.16 (.85)
TMT: interference indicator (z-scores)	.88 (1.46)	–.24 (.61)
TMT-A: errors (z-scores)	.32 (1.42)	–.20 (0.45)
TMT-B: errors (z-scores)	.90(1.69)	–.30(.36)
SCWT: interference effect (errors) (z-scores)	.34(1.20)	–.15(.73)
SCWT: interference effect (time) (z-scores)	.71(1.31)	–.18(.79)

*EF* executive functions, *CPT* continuous performance task, *TMT* trail making test A and B, *SCWT* Stroop Color Word Interference Test.

Adjusted analysis showed the odds of being overfat increased nearly threefold (95% CI [1.562, 5.175], *p* < 0.001) and the odds of being obese-fat increased more than threefold (95% CI [1.656, 6.687], *p* < 0.001) for children with higher error interference effect in SCWT. However, the risk decreased by nearly 50% (respectively for overfatness and fat-obesity 95% CI [0.254, 0.836], *p* = 0.011; 95% CI [0.213, 0.947], *p* = 0.035) for children with more commission errors in CPT. In the unadjusted analysis, more errors in TMT-B increased the odds of being excess weight being (95% CI [1.532, 6.898], *p* = 0.002), however, the association lost its significance after the analysis was adjusted analysis with controlled variables. The detailed results of logistic regression analyses are presented in Table S4, which is available in the supplementary materials. The results of the r Pearson correlation showed small correlations between some of the SCWT and the CPT results and FMI and BF (%) z-scores. It was also observed thatthe higher interference effect in the SCWT, the higher level of body fat (%) in children (r = 0.152, *p* < 0.01) and the higher FMI z-score in children (r = 0.116, *p* < 0.05). Furthermore, the longer reaction time in CPT, the higher BF (%) (r = 0.097, *p* < 0.05) and FMI z-scores (r = 0.097, *p* < 0.05) in the children. The effect was not observed for BMI, regardless of the EF indicator (*p* > 0.05).

### The FTO genotype and cool EFs

The genotyping data were available for 458 children. The distribution of the observed genotypes followed the Hardy–Weinberg equilibrium (*p* = 0.177). In the sample the allele frequency was 44.54% and 55.46% for allele A and allele T respectively. Based on the adjusted logistic regression analysis, it was observed that the odds of lower executive functioning increased over 2.5-fold with having the FTO risk allele (AA/AT vs TT, 95% CI [1.205, 5.226], *p* = 0.014; AA vs TT, 95% CI [1.046, 6.449], *p* = 0.040). However, further analyses for detailed indicators of EFs showed no association between most of the EFs and the FTO genotype. The only significant result was obtained for the association between errors in TMT-B and the AA homozygote. The odds of making more errors in TMT-B was over threefold (95% [1.043, 12.443], *p* = 0.043) higher in the AA homozygotes in comparison to other FTO genotypes. The results of the logistic regression analyses are presented in Table 3 below.

**Table 3:** The results of logistic regression in unadjusted and adjusted analyses for cool EFs (dependent variable) according to FTO genotype (independent variable).

	AA/AT vs TT (reference: TT)				AA vs TT (reference: TT)			
	Unadjusted		Adjusted		Unadjusted		Adjusted	
	OR (95% CI)	*p*	OR (95% CI)	*p*	OR (95% CI)	*p*	OR (95% CI)	*p*
Cool EFs: overall (clusters) (reference: better results)	**1.911 (1.064–3.434)**	**.030**	**2.509 (1.205–5.226)**	**.014**	1.802 (.870–3.732)	.113	**2.599 (1.046–6.449)**	**.040**
CPT: reaction time (reference: faster RT)	.928 (.612–1.408)	.727	.756 (.476–1.203)	.238	.921 (.533–1.589)	.767	.805 (.433–1.499)	.494
CPT: commission errors (reference: less errors)	.994 (.603–1.641)	.982	.900 (.507–1.598)	.720	.911 (.468–1.772)	.783	.769 (.348–1.701)	.517
CPT: omission errors (reference: less errors)	.928 (.558–1.542)	.773	.757 (.429–1.334)	.335	.842 (.428–1.660)	.620	.650 (.297–1.425)	.282
TMT: interference effect (reference: lower effect)	1.227 (.638–2.359)	.540	1.672 (.788–3.548)	.180	1.316 (.581–2.980)	.511	1.816 (.710–4.650)	.213
TMT-A: errors (reference: less errors)	1.329 (.546–3.235)	.531	2.724 (.770–9.642)	.120	1.790 (.628–5.108)	.276	3.464 (.813–14.751)	.093
TMT-B: errors (reference: less errors)	1.424 (.649–3.121)	.378	2.732 (.915–8.157)	.072	1.568 (.599–4.103)	.359	**3.603 (1.043–12.443)**	**.043**
SCWT: interference effect—errors (reference: lower effect)	.958 (.476–1.927)	.903	.748 (.350–1.598)	.453	.690 (.252–1.887)	.469	.654 (.227–1.890)	.433
SCWT: interference effect—time (reference: lower effect)	1.217 (.795–1.862)	.365	1.138 (.711–1.822)	.590	.889 (.503–1.570)	.685	.810 (.423–1.551)	.525

*EFs* executive functions, *CPT* continuous performance task, *TMT* trail making test A and B, *SCWT* Stroop Color Word Interference Test, *BMI* body mass index, *FMI* fat mass index,* p* significance level, *bold* significant difference, *n* number of individuals in the categories.

All analyses are adjusted for: mother’s education (reference: higher education), father’s education (reference: higher education), parental assessment of family’s socioeconomic status (reference: good or very good), place of residence (reference: large city).

Furthermore, there were no differences between the children’s neuropsychological performance in the ANOVA analysis. Detailed results are presented below in Table 4.

**Table 4:** The differences in children’s neuropsychological parameters according to FTO genotype (AA, AT, TT) (ANOVA).

	AA		AT		TT		ANOVA results		
	n	Mean (SD)	n	Mean (SD)	n	Mean (SD)	F	df	*p*
CPT: reaction time (z-scores)	97	− .01 (1.01)	211	− .01 (1.07)	148	− .02 (.90)	.004	2	.996
CPT: commission errors (z-scores)	97	.07 (.85)	211	.04 (1.02)	148	− .03 (.98)	.406	2	.667
CPT: omission errors (z-scores)	94	− .01 (.98)	199	.04 (1.02)	143	− .03 (.94)	.219	2	.804
TMT: interference effect (z-scores)	98	− .10 (.76)	206	− .03 (.89)	146	− .03 (1.14)	.228	2	.796
TMT-A: errors (z-scores)	98	.09 (1.09)	209	− .08 (.78)	148	− .05 (.90)	1.224	2	.295
TMT-B: errors (z-scores)	97	.14 (1.29)	209	.01 (1.06)	147	− .10 (.78)	1.669	2	.190
SCWT: interference effect—errors (z-scores)	89	− .11 (.65)	196	− .05 (.95)	137	− .07 (.77)	.136	2	.873
SCWT: interference effect—time (z-scores)	89	− .15 (.84)	196	.10 (1.01)	138	.00 (1.03)	2.072	2	.127

*CPT* continuous performance task, *TMT* trail making test A and B, *SCWT* Stroop Color Word Interference Test, *BMI* body mass index, *FMI* fat mass index, *F* Fisher’s exact test, *n* number of individuals in the categories, *df* degrees of freedom, *p* significance level.

Then, the results of the factorial ANOVA showed no significant interaction between the FTO genotype (AA, AT, TT) and the cool EFs (*p* = 0.350). The results are presented below in Table 5.

**Table 5:** The effects of interaction between the FTO genotype and cool EFs (clusters) according to the BMI and FMI z-scores.

	BMI z-scores					FMI z-scores				
	SS	df	MS	F	*p*	SS	df	MS	F	*p*
Intercept	17.095	1	17.096	11.667	**.000**	2.476	1	2.476	2.981	.085
Cool EFs (clusters)	.361	1	.361	.247	.620	.589	1	.589	.709	.400
FTO genotype (AA, AT, TT)	2.395	2	1.197	.817	.442	2.014	2	1.007	1.212	.298
Cool EFs (clusters) × FTO genotype (AA, AT, TT)	3.082	2	1.541	1.052	.350	1.193	2	.596	.718	.488

*EFs* executive functions, *SS* sum of squares, *df* degrees of freedom, *MS* mean square, *F* Fisher's exact test, *p* significance level, *bold* significant effect (*p* < .05).

The factorial ANOVA results.

### Controlled variables

In the unadjusted analyses the association of each controlled variable, but the place of residence, and the children’s body mass and fatness was found to be significant. The biggest effects were observed for parental education. The odds of having excess weight increased more than twofold (95% CI [1.146, 3.363], *p* < 0.001) and over six fold (95% CI [2.655, 15.721], *p* < 0.001) when a mother did not have higher education, as well as increased consecutively twofold (95% CI [1.398, 3.456], *p* < 0.001) and fourfold (95% CI [1.636, 12.144], *p* = 0.003) in children with low educated fathers. The risk of being obese-fat was 1.5 times higher in children with low education both mother (95% CI [1.116, 2.734], *p* = 0.015) and father (95% CI [1.065, 2.656], *p* = 0.026). The effect was not observed for the risk of being overfat. Furthermore, there were found significant associations between the parental assessment of a family’s SES, mother’s body mass status and children’s body mass status. The bad or very bad parental assessment of family’s SES increased the odds of being overweight (95% CI [1.065, 2.867], *p* = 0.027) or obesity in children (95% CI [1.104, 5.751], *p* = 0.028). Finally, the risk of being overweight or obese increased more than twofold (95% CI [1.554, 3.838], *p* < 0.001) and nearly fivefold (95% CI [2.096, 10.741], *p* < 0.001) with a mother’s excess weight. The effect was observed as well for overfatness (95% CI [1.035, 2.286], *p* = 0.033) but not for fat obesity (*p* > 0.05). Detailed results are presented below in Table S2 in the Supplement.

Finally, we obtained interesting results by including the variables controlled for body mass in a single model with the FTO genotype polymorphism and cool EFs clusters. In this model, maternal BMI was the only variable that increased the risk of children’s being overweight (95% [1.257–4.139], *p* = 0.007), and maternal education was the only variable that increased the risk of obesity diagnosed according to the IOTF standards (95% CI [1.1931–38.183], *p* = 0.005) and obesity according to the McCarthy criteria (95% CI [1.566–8.876], *p* = 0.003).Detailed results are presented below in Table S3 in the Supplement.

## Discussion

The aims of the study were to assess the association of the cool executive functions with body mass & fatness, and to assess the role of the FTO gene polymorphism in this possible relationship in school-aged children. The study revealed a partial association of cool EFs with body fatness. In turn, the results of the relationship between the FTO genotype and EFs delivered ambiguous results.

### Executive functions, body mass and body fatness

In our own study, we found a significant association between the error interference effect in SCWT and body fatness. The interference effect in SCWT is thought to be the main indicator in a SCWT task for the inhibitory control function^38^. Thus, we assessed that the inhibitory control was presumably the EF with the strongest associations with body mass & fat indicators in our study. In the previous research, cognitive control has been most strongly documented as an EF function associated with overweight and obesity^27,28^. The present study confirmed the research trend to date. Although, EFs do not operate independently of each other, inhibitory control is particularly thought to be relevant to the processes of self-control and self-regulation^14^. It has been suggested that an appropriate level of self-control allows for optimal dietary choices through the better control of appetite and responding to satiety signals even with the high tastiness of the food consumed^39^. Thereby influencing the improvement of EFs, including inhibitory control^40^, individual differences and potential deficits may be significant for the development of obesity as early as this period of life^41^. Interestingly, an increased number of commission errors in CPT, which is an indicator of impulsivity, decreased the risk of excess fatness in adjusted analyses. Nevertheless, taking into consideration that another analysis did not reveal the associations of commission errors and body mass and fatness indicators, the result should be interpreted with caution.

Sustained attention, considered as a component of EFs^14^, and shifting or cognitive flexibility are also thought to be engaged in the body mass control process^17,18^. According to Dohle et al.^20^, switching deficits are associated with an extremely low or high BMI but there is limited evidence in this field in non-clinical samples. In our study the relationships between shifting, measured by the interference effect in TMT, and overweight or obesity, as well as with body fatness were insignificant in the adjusted analyses. The role of switching in the eating behavior regulation may be moderating^20^. The relationship can manifest as mental flexibility in the choice of behavior to achieve a dietary goal and the ability to use alternatives^42^. We did not examine neither of the children’s eating behaviours nor the children’s eating attitudes, thus, we only can conclude about the direct relationship between shifting and body mass. Finally, some of the previous studies suggest the possible role of poor sustained attention in developing increased body mass in adults^43^. In turn, in our study we did not find sustained attention is associated with excess weight or excess body fat in children.

The area worth discussing is why we obtained an association mostly with body fatness and not with body mass. First, at this developmental stage it might be too early for the EF-obesity effect to manifest itself. By the time obesity is diagnosed with a BMI, excess body fat may already be apparent because of an unhealthy diet and less active lifestyle. Thus, it is possible to be characterized by a healthy body weight and excessive body fat at the same time. An excessive amount of body fat despite healthy body weight has been described in the literature as *normal-weight obesity syndrome* (NWO)^44^. In our study, there were significantly more children with excessive body fat diagnosed based with the McCarthy criteria^33^ compared to those with excessive body weight diagnosed and based on the IOTF criteria. Over ¼ of healthy weight children were characterised by over-fatness. Thus, the body fat indicator could have been more sensitive to an obesogenic lifestyle than BMI.

On the other hand, our study did not examine causality, only correlations. Therefore, we cannot exclude the possibility that it was excessive fatness that adversely affected the inhibitory control function. Excessive levels of body fat are associated with an increased level of pro-inflammatory cytokines (e.g., interleukin 1, interleukin 6) that can cross the blood–brain barrier and impair synaptic neuroplasticity and cognitive functioning in turn^45^. A two-way EF—body fatness relationship is likewise not excluded.

Our hypothesis about the relationship of the FTO gene with cool EFs in children was novel. To our knowledge, our study is the first to test the association of the FTO gene with cognitive functioning in children in the context of obesity. Our analysis does not reveal a clear line of trend. Most of the results suggest no association between the FTO genotype and specific executive functions in the studied children. However, considering overall executive functioning, an increased risk of lower EFs was shown for carriers of the A allele in adjusted analyses, suggesting the risk for obesity allele might be associated as well with the risk of EF impairment. However, concurrently, we did not demonstrate a potential interaction between FTO genotype, executive functions, and the risk of excessive weight in children. It is possible that this relationship is not strong enough during childhood to manifest itself at a more visible level. This brings us to the consideration of the role of environmental factors in the widespread child development.

The analyses of the relationship between environmental factors, such as the socioeconomic status of the family or both maternal and father education, revealed a strong significant role of these factors on the children’s excess weight development. These factors had a markedly stronger effect on obesity development than the cool EFs. The strongest predictors of excessive body mass and fatness in our study was parental education. Although both the mother's and father's low education increased the risk of excessive weight in children, the child's risk of obesity was the highest when the mother lacked higher education. Maternal education remained a statistically important factor in the analyses that included cool EFs and the FTO genotype as well. These results may be explained by a higher nutrition knowledge and an awareness of developing good eating habits among educated parents^46,47^. Furthermore, parents with an education beyond high school level spend more on family healthcare^48^, so they can respond to early signals of developing health difficulties, including excessive weight. Considering that better executive functioning is associated with higher academic achievements and higher education^49^, in this context the area worthy of discussion is the potential effect of maternal EFs on the organisation of a family’s diet and in turn, on the children’s body mass. As far as we know, there is a research gap in this area. However, the work of Bauer and colleagues^50^ suggests that the lower EFs of mothers may be related to a less healthy home food environment, including difficulties with providing frequent family meals and more often using food as a regulator of child’s emotions in comparison to mothers with high executive functioning. Conceivably, mothers with lower education may be characterized by lower levels of EFs and thus have more difficulty organizing meals in a consistent, healthy manner.

In turn, the higher impact of low maternal rather than the father’s education on the obesity pf the children in our study might be associated with the family structure in Poland. Although social roles in Poland are changing from a patriarchal model to egalitarian model^51^, traditional gender roles, such as the maternal care of diet and meal preparing, still have a prominent role in the country^52^. Finally, alluding to the negative assessment of the family's economic situation, this factor may increase the risk of overweight and obesity in the children due to the lack of available funds for private medical care or better-quality food.

### Strength, limitations, and further research directions

One of the study’s strengths was that we considered additional indicators of overweight and obesity unlike most studies on the EF-body mass association: not only BMI, but FMI and body fatness (%) as well. We studied cool EFs with three different methods, which provided a wider field for analysis. We further put a novel hypothesis about the relationship between the FTO gene and executive functions in children.

However, some limitations should be taken into consideration. First, the study is of a cross-sectional nature. Thus, we can analyze the differences and the associations of cool EF—body mass/body fat link but not the causation. Secondly, despite the preliminary instructions for subjects’ parents before the study, there was no control over the children’s food and water consumption on the day of measurement included in the examination procedure. That might have had an impact on the body composition analyses results. However, it should not be clinically significant^53^. Due to the exploratory nature of the study, the results were not corrected for multiple testing. Therefore, while the risk of making a Type II error is limited, the risk of making a Type I error is greater. Caution should therefore be maintained during the interpretation of the results. Further limitations relate to neuropsychological tools. The Stroop Color-Word Interference Test was done on paper and not on a computer. This potentially increased the risk of researcher mistakes. In addition, we did not get as detailed reaction time data as would have been possible in a computerized measurement. However, the tool we chose has been used successfully in other studies in Poland^54^. Then, both the SCWT and TMT test were found to be too difficult for children in the youngest age groups, thus other versions of neuropsychological tasks should be taken into consideration for younger groups. This resulted in a smaller study group for analysis. Furthermore, the participants in the study were mostly from educated families, which could have biased the results. Finally, our sample included children without any clinical psychiatric diagnoses and mental illness. Thus, strong EF deficits might have been difficult to observe. Nevertheless, the aim of our study was to study the association of cool EFs and BMI/adiposity in typically developed children. Further research directions in our opinion should focus on exploring the relationship between EFs and dietary goal-oriented behaviours, not only between investigating EFs and body mass indicators. Furthermore, in order to study the influence EFs on body mass, the effectiveness of cognitive training and supporting self-regulation skills on the ability to adequately control nutrition should be tested. An interesting research direction might be as well studying the association between a mother’s EFs, children’s eating behaviours and body mass or body fatness. Finally, further studies on the common genetic background of EFs and obesity are worth exploring.

### Conclusions

Considering all our results on the association of cool EFs with body mass in neurotypically developed children, we do not find a conclusive trend of the studied relationship, except for the small effect of inhibitory control on increased body fatness. We can consider that the tendency might be marked as a prominent relationship in the later stages of ontogenies when there is a greater autonomy of individuals in dietary choices^55^. Likewise, there remained no explicit association between the FTO genotype and executive deficits in children. We believe that during childhood, it is the family environment that plays a crucial role in the prevention of being overweight or obese in children.

## Supplementary Information

Supplementary Information.

## Data Availability

The datasets generated during and/or analysed during the current study are available from the corresponding author on reasonable request.
