# Supplementary material for: Cool executive functions and their association with body mass & fatness and the FTO gene in school-aged children
Source: Sci Rep. 2023 Jul 27;13:12148. doi: 10.1038/s41598-023-38808-0 (PMC10374888; doi:10.1038/s41598-023-38808-0)
Supplement: Supplementary file 1 — Supplementary Information. [file 41598_2023_38808_MOESM1_ESM.docx]

**Table S1. Neuropsychological results according to age and sex**

|  |  | Test results | | | | | | | | | | |
| --- | --- | --- | --- | --- | --- | --- | --- | --- | --- | --- | --- | --- |
|  |  | CPT: | | | | TMT: | | | | SCWT | | |
|  |  |  | reaction time [ms] | commision errors | ommision errors |  | Part A: errors | Part B: errors | Interfrence rate |  | Time interference | Error interference |
|  | Age | N | Mean (SD) | Mean (SD) | Mean (SD) | N | Mean SD) | Mean (SD) | Mean (SD) | N | Mean (SD) | Mean (SD) |
| Girls | 6y | 3 | 976.66 (105.07) | 60.10 (42.14) | 18.50 (12.02) | 0 | - | - | - | 0 |  |  |
|  | 7y | 30 | 998.00 (126.60) | 44.16 (35.36) | 15.41 (6.26) | 27 | 5.7 (1.28) | 3.86 (5.49) | 4.29 (2.04) | 25 | 78.28 (33.92) | 1.64 (3.13) |
|  | 8y | 76 | 906.05 (152.91) | 30.07 (32.91) | 13.62 (9.59) | 75 | .18 (.764) | 3.91 (18.11) | 3.48 (1.25) | 66 | 70.91 (40.61) | 1.33 (2.09) |
|  | 9y | 72 | 919.02 (241.31) | 27.63 (21.12) | 11.42 (10.31) | 72 | .09 (.334) | .78 (1.45) | 3.78 (3.28) | 72 | 63.19 (30.29) | 1.08 (2.10) |
|  | 10y | 48 | 825.75 (101.90) | 34.21 (33.46) | 8.69 (5.75) | 48 | 2.77 (20.47) | .93 (2.88) | 3.35 (1.23) | 48 | 59.04 (25.85) | 1.02 (2.09) |
|  | 11y | 19 | 788.21 (97.17) | 34.21 (33.46) | 7.27 (5.20) | 20 | .00 (.00) | 7.90 (34.85) | 2.94 (.86) | 20 | 52.55 (22.41) | 1.10 (1.99) |
|  | 12y | 6 | 775.50 (98.77) | 14.66 (15.82) | 4.00 (1.26) | 6 | .08 (.289) | 2.75 (6.77) | 3.69 (1.16) | 6 | 33.00 (12.71) | .00 (.00) |
| Boys | 6y | 0 | - | - | - |  | - | - | - |  | - | - |
|  | 7y | 32 | 940.66 (169.19) | 74.34 (123.46) | 15.50 (9.62) | 32 | 7.9 (1.97) | 1.50 (3.27) | 3.94 (1.94) | 19 | 79.95 (34.61) | 2.53 (2.84) |
|  | 8y | 80 | 887.41 (213.85) | 52.02 (46.94) | 52.02 (46.94) | 79 | 2.31 (18.46) | 4.52 (18.59) | 3.79 (1.76) | 73 | 64.79 (36.40) | 1.21 (2.75) |
|  | 9y | 70 | 855.36 (212.17) | 54.41 (62.07) | 11.20 (9.49) | 70 | 2.11 (17.99) | 5.99 (25.25) | 3.48 (1.26) | 69 | 65.71 (29.96) | 1.81 (4.12) |
|  | 10y | 53 | 813.60 (210.24) | 29.51 (25.93) | 10.80 (11.22) | 53 | .11 (4.16) | 1.13 (2.45) | 3.34 (1.42) | 53 | 50.42 (28.44) | 1.21 (2.05) |
|  | 11y | 31 | 828.55 (136.28) | 35.52 (39.67) | 9.00 (5.79) | 31 | 4.63 (27.03) | 5.51 (26.99) | 3.72 (1.13) | 31 | 52.97 (23.91) | 1.19 (2.07) |
|  | 12y | 17 | 716.23 (208.50) | 27.53 (21.92) | 11.81 (14.38) | 17 | .08 (.277) | .76 (1.715) | 4.06 (1.60) | 17 | 44.71 (15.20) | .53 (1.59) |

CPT – Continuous Performance Task, RT – reaction time, TMT – Trail Making Test A and B, SCWT – Stroop Color Word Interference Test

**Table S2. The results of logistic regression in unadjusted analyses for controlled variables**

|  | Healthy weight vs excess weight (IOTF criteria) | | Healthy fat vs overfat (McCarthy criteria) | | Healthy weight vs obesity (IOTF criteria) | | Healthy fat vs obesity (McCarthy criteria) | |
| --- | --- | --- | --- | --- | --- | --- | --- | --- |
|  | OR (95% CI) | p | OR (95% CI) | p | OR (95% CI) | p | OR (95% CI) | p |
| Mother’s BMI (reference: healthy body mass) | **2.442 (1.554-3.838)** | **<.001** | **1.538 (1.035-2.286)** | **.033** | **4.745 (2.096-10.741)** | **<.001** | 1.585 (.976-2.572) | .062 |
| Mother’s education (reference: higher education) | **2.182 (1.146-3.363)** | **<.001** | 1.266 (.872-1.838) | .215 | **6.461 (2.655-15.721)** | **<.001** | **1.747 (1.116-2.734)** | **.015** |
| Father’s education (reference: higher education) | **2.198 (1.398-3.456)** | **<.001** | 1.262 (.876-1.818) | .212 | **4.458 (1.636-12.144)** | **.003** | **1.682 (1.065-2.656)** | **.026** |
| Parental assessment of family’s SES (reference: good or very good) | **1.747 (1.065-2.867)** | **.027** | 1.256 (.811-1.944) | .307 | **2.520 (1.104-5.751)** | **.028** | 1.256 (.738-2.140) | .401 |
| Place of residence (reference: large city) | 1.012 (.587-1.745) | .966 | .919 (.584-1.1445) | .713 | 1.200 (.465-3.099) | .706 | 1.162 (.683-1.986) | .580 |

**bold** – significant difference (p.<05); *p* – significance level

**Table S3. The results of logistic regression in adjusted and unadjusted analyses for body mass and fatness according to FTO genotype and controlled factors**

|  |  | Healthy weight vs excess weight (IOTF criteria) | | Healthy fat vs overfat (McCarthy criteria) | | Healthy weight vs obesity (IOTF criteria) | | Healthy fat vs obesity (McCarthy criteria) | |
| --- | --- | --- | --- | --- | --- | --- | --- | --- | --- |
|  |  | OR (95% CI) | p | OR (95% CI) | p | OR (95% CI) | p | OR (95% CI) | p |
| *Unadjusted analysis* | |  |  |  |  |  |  |  |  |
|  | FTO (AA/AT vs TT) (reference: TT) | .980 (.601-1.598) | .935 | 1.049 (.684-1.609) | .825 | 1,776 (.645-4.892) | .267 | 1.111 (.632-1.952) | .715 |
| *Adjusted analysis* | |  |  |  |  |  |  |  |  |
|  | FTO (AA/AT vs TT) (reference: TT) | .634 (.342-1.176) | .149 | .829 (.483-1.421) | .494 | 1.099 (.343-3.522) | .874 | .877 (.396-1.944) | .747 |
|  | Cool EFs: overall (clusters) (reference: better results) | 1.240 (.581-2.648) | .579 | 1.008 (.520-1.952) | .981 | .998 (.249-4.007) | .998 | .559 (.196-1.595) | .277 |
|  | Mother’s BMI (reference: healthy body mass) | **2.272 (1.247-4.139)** | **.007** | 1.146 (.660-1989) | .628 | 2.005 (.675-5.960) | .211 | 1.122 (.512-2.460) | .773 |
|  | Mother’s education (reference: higher education) | 1.518 (.783-2.943) | .217 | 1.763 (.964-3.223) | .066 | **8.587 (1.931-38.183)** | **.005** | **3.728 (1.566-8.876)** | **.003** |
|  | Father’s education (reference: higher education) | 1.646 (.842-3.215) | .145 | 1.214 (.671-2.197) | .522 | 1.340 (.339-5.300) | .676 | 1.910 (.757-4.820) | .171 |
|  | Parental assessment of family’s SES (reference: good or very good) | 1.299 (.652-2.588) | .457 | 1.221 (.663-2.247) | .522 | .418 (.085-2.057) | .283 | 1.028 (.428-2.469) | .950 |
|  | Place of residence (reference: large city) | 1.409 (.716-2.776) | .321 | 1.137 (.625-2.070) | .674 | 1.223 (.344-4.342) | .756 | 1.754 (.779-3.952) | .175 |

EFs – executive functions, **bold** – significant difference (p.<05); *p* – significance level

**Table S4. The results of logistic regression in adjusted and unadjusted analyses for body mass and fatness according to cool EFs’ indicators**

|  | Healthy weight vs excess weight (IOTF criteria) | | | | Healthy fat vs overfat (McCarthy criteria) | | | | Healthy weight vs obesity (IOTF criteria) | | | | Healthy fat vs obesity (McCarthy criteria) | | | |
| --- | --- | --- | --- | --- | --- | --- | --- | --- | --- | --- | --- | --- | --- | --- | --- | --- |
|  | Unadjusted | | Adjusted | | Unadjusted | | Adjusted | | Unadjusted | | Adjusted | | Unadjusted | | Adjusted | |
|  | OR (95% CI) | p | OR (95% CI) | p | OR (95% CI) | p | OR (95% CI) | p | OR (95% CI) | p | OR (95% CI) | p | OR (95% CI) | p | OR (95% CI) | p |
| Cool EFs: overall (clusters) (reference: better results) | 1.420 (.810-2.487) | .221 | 1.122 (.569-2.215) | .739 | 1.328 (.801-2.202) | .271 | 1.092 (.601-1.984) | .774 | 1.703 (.634-4.580) | .291 | 1.460 (.380-5.607) | .581 | 1.230 (.623-2.426) | .551 | 1.986 (.779-5.064) | .151 |
| CPT: reaction time (reference: faster RT) | 1.048 (.670-.1650) | .837 | 1.196 (.717-1.996) | .493 | 1.260 (.866-1.833) | .227 | .880 (.581-1.334) | .548 | 1.403 (.632-3.117) | .405 | 1.055 (.418-2.660) | .910 | 1.306 (.826-2.064) | .254 | .875 (.522-1.466) | .611 |
| CPT: commission errors (reference: less errors) | 1.251 (.734-2.131) | .410 | .918 (.483-1.743) | .793 | .639 (.390-1.046) | .075 | **.461 (.254-.836)** | **.011** | 2.038 (.854-4.863) | .108 | .980 (.326-2.941) | .971 | .778 (.434-1.396) | .401 | **.449 (.213-.947)** | **.035** |
| CPT: omission errors (reference: less errors) | 1.488 (.861-2.574) | .155 | 1.013 (.536-1.915) | .969 | 1.102 (.670-1.815) | .701 | .854 (.480-1.520) | .592 | 1.667 (.630-4.411) | .303 | .822 (.248-2.720) | .748 | 1.556 (.850-2.850) | .152 | .956 (.454-2.012) | .906 |
| TMT: interference effect (reference: lower effect) | .764 (.391-1.490) | .429 | .716 (.337-1.519) | .383 | 1.098 (.650-1.857) | .726 | 1.174 (.662-2.082) | .582 | .779 (.226-2.683) | .779 | .515 (.110-2.398) | .397 | 1.450 (.794-2.648) | .226 | 1.523 (.776-2989) | .221 |
| TMT-A: errors (reference: less errors) | 1.175 (.510-2.706) | .704 | .796 (.278-2.280) | .671 | 1.190 (.591-2.399) | .626 | .890 (.384-2.064) | .785 | 1.261 (.281-5.668) | .762 | .490 (.057-4.199) | .515 | 1.241 (.533-2.891) | .617 | .689 (.222-2.141) | .519 |
| TMT-B: errors (reference: less errors) | **3.251 (1.532-6.898)** | **.002** | 2.464 (.974-6.232) | .057 | 1.184 (.602-2.328) | .625 | .843 (.367-1.935) | .687 | 1.918 (.416-8.854) | .404 | .856 (.096-7.619) | .889 | 1.162 (.503-2.683) | .725 | .655 (.212-2.019) | .461 |
| SCWT: interference effect – errors (reference: lower effect) | 1.592 (.856-2.959) | .142 | 1.615 (.814-3.202) | .170 | **2.753 (1.577-4.807)** | **<.001** | **2.843 (1.562-5.175)** | **<.001** | 1.098 (.314-3.842) | .884 | 1.368 (.350-5.352) | .653 | **2.920 (1.528-5.581)** | **.001** | **3.328 (1.656-6.687)** | **<.001** |
| SCWT: interference effect – time (reference: lower effect) | 1.042 (.660-1.645) | .860 | 1.007 (.596-1.702) | .979 | 1.225 (.835-1.796) | .299 | 1.095 (.711-1.685) | .681 | .839 (.352-1.999) | .691 | .865 (.320-2.338) | .775 | 1.054 (.653-1.701) | .830 | .907 (.525-1.566) | .726 |

EFs – executive functions, CPT – Continuous Performance Task, RT – reaction time, TMT – Trail Making Test A and B, SCWT – Stroop Color Word Interference Test, OR – an odds ratio in logistic regression analysis, *p* – significance level**; bold** - significant difference (p<.05)
